# The Level and Duration of RSV-Specific Maternal IgG in Infants in Kilifi Kenya

**DOI:** 10.1371/journal.pone.0008088

**Published:** 2009-12-02

**Authors:** Rachel Ochola, Charles Sande, Gregory Fegan, Paul D. Scott, Graham F. Medley, Patricia A. Cane, D. James Nokes

**Affiliations:** 1 Kenya Medical Research Institute, Centre for Geographic Medicine Research-Coast, Kilifi, Kenya; 2 Department of Epidemiology & Population Health, London School of Hygiene and Tropical Medicine, London, United Kingdom; 3 Department of Biological Sciences, University of Warwick, Coventry, United Kingdom; 4 Health Protection Agency, London, United Kingdom; Singapore Immunology Network, Singapore

## Abstract

**Background:**

Respiratory syncytial virus (RSV) is the major cause of lower respiratory tract infection in infants. The rate of decay of RSV-specific maternal antibodies (RSV-matAb), the factors affecting cord blood levels, and the relationship between these levels and protection from infection are poorly defined.

**Methods:**

A birth cohort (n = 635) in rural Kenya, was studied intensively to monitor infections and describe age-related serological characteristics. RSV specific IgG antibody (Ab) in serum was measured by the enzyme linked immunosorbent assay (ELISA) in cord blood, consecutive samples taken 3 monthly, and in paired acute and convalescent samples. A linear regression model was used to calculate the rate of RSV-matAb decline. The effect of risk factors on cord blood titres was investigated.

**Results:**

The half-life of matAb in the Kenyan cohort was calculated to be 79 days (95% confidence limits (CL): 76–81 days). Ninety seven percent of infants were born with RSV-matAb. Infants who subsequently experienced an infection in early life had significantly lower cord titres of anti-RSV Ab in comparison to infants who did not have any incident infection in the first 6 months (*P = 0.011*). RSV infections were shown to have no effect on the rate of decay of RSV-matAb.

**Conclusion:**

Maternal-specific RSV Ab decline rapidly following birth. However, we provide evidence of protection against severe disease by RSV-matAb during the first 6–7 months. This suggests that boosting maternal-specific Ab by RSV vaccination may be a useful strategy to consider.

## Introduction

Respiratory syncytial virus (RSV) is the single most important viral cause of lower respiratory tract infection (LRTI) during infancy and early childhood worldwide [1], [2], [3]. The role of RSV antibodies (Ab) in infant protection in the developing world, where the epidemiology of RSV and related infections may differ from that in developed regions, is only now being evaluated [2], [4]. There is a paucity of information on the role of RSV-specific maternal antibodies (RSV-matAb).

The incidence of bronchiolitis in infants below 2 months of age has been observed to be markedly lower than in older children [5], [6], [7], [8]. The incidence of pneumonia has also been documented as being lower but less strikingly so. Additionally, pneumonia is uncommon and bronchiolitis very rare in infants under 3 weeks of age [9], [10]. Within this context therefore, RSV-matAb play an important role in protection against severe RSV-associated illness. While there is evidence that RSV-matAb are likely to be important in protection against RSV disease, severe disease is noted in children under 6 months of age, paradoxically at a time when RSV-matAb are frequently identified, but may be waning.

The importance of RSV-matAb protection against disease is further illustrated by the fact that infants born during or just prior to the RSV season, during the time when titers are likely to be the lowest in the mothers, have been described as being at maximum risk of admission to hospitals with RSV infection in the ensuing RSV season [11]. Further studies have shown the relationship of neutralizing Ab titres to RSV with severity of LRTI, noting an inverse correlation between the two measures [2], [8], [12], [13], [14], [15]. These observations underscore the importance of high matAb concentration in reducing the risk of severe disease in early months of life.

It has also been demonstrated that the administration of prophylactic intravenous immunoglobulin enriched for high levels of RSV neutralizing Ab (RSV Immune Globulin or RSV-IGIV) or humanized monoclonal Ab against F protein are associated with reduction of RSV-associated disease [12], [16], [17]. The use of maternal immunization to augment this protection in young infants against disease shows promise and thus lends itself to further consideration [18]. It is plausible that RSV-associated disease under 6 months of age is due to low RSV-matAb levels and/or RSV-matAb not being fully protective (for example, due to strain-specificity). The latter have also been said to interfere with vaccine efficacy and may eventually lead to vaccine failure [19] especially with regards to early paediatric live vaccines.

To document the levels and duration of RSV-matAb, and to understand factors which may affect these levels, we undertook to characterize the levels of RSV-matAb and their decay rate in a birth cohort from rural Kenya. The effects of risk factors on matAb decay and cord titres were investigated using multiple linear regression analysis. This work is preliminary to further analysis of the relationship between matAb level and protection.

## Materials and Methods

### Ethics Statement

The research project was one of several studies nested within a birth cohort study of RSV [20], [21] in Kilifi District, which was reviewed and passed as ethically acceptable by the Kenya Medical Research Institute/National Ethic Review Committee and Coventry Research Ethics Committee, UK. Written informed consent was obtained from each caregiver of every child enrolled in the study.

### Study Population and Samples

The study was carried out in the District of Kilifi an area covering 4779 km^2^ in rural coastal Kenya. The population of around 500,000, predominantly subsistence farmers, has a growth rate of 3.1% per annum, with 18%<5 years in age [22]. The District hospital is located within Kilifi town, located 60 km north of Kenya's main port city of Mombasa.

**Figure 1:**
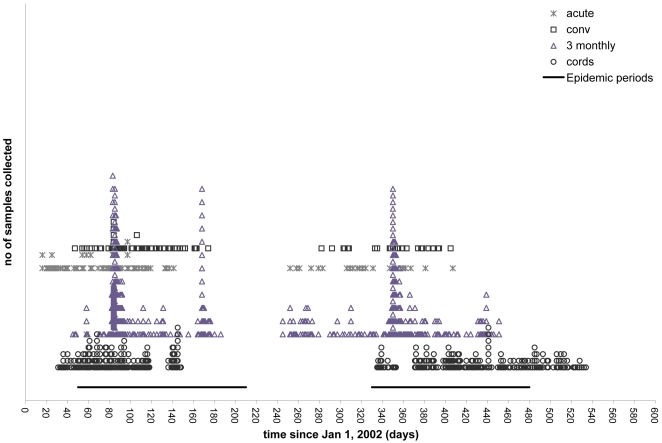
Schema of sample collection regime for Kilifi cohort. 635 children were recruited to a birth cohort in 2 phases (February- May 2002, and December 2002- May 2003). Cord bloods (circles), together with sera every 3 months (triangles) were collected. Any antigen positive nasal washing detected by IFAT collected upon each ARI episode, prompted collection of paired acute (asterix) and convalescent (squares) phase sera. The first 2 epidemics experienced by the cohort are indicated by the black bars.

Newly delivered babies at Kilifi District Hospital (KDH) or infants attending the Maternal Child Health Clinic at KDH within the first 2 weeks of life were recruited. About 300 children each were recruited in 2 phases to the birth cohort, between February – May 2002, and December 2002 – May 2003 respectively, and were intensively monitored for acute respiratory infections (ARI). The surveillance procedure of infants, including the details of collection, cold chain to the laboratory and sample processing has been extensively described previously [20], [21]. Briefly, active household surveillance for ARI was weekly during RSV epidemics and monthly otherwise. Passive surveillance was carried out principally through parental referral to outpatient research clinic (Monday- Friday, 8 A.M- 5 P.M.) at KDH [21]. Passive referral was encouraged for a child with any ARI symptoms. At each contact, a nasal washing (NW) was collected if the child had any respiratory symptoms. Blood samples, which included cord blood and repeated blood samples taken at approximately 3 monthly intervals were collected from each child; if a child experienced a positive RSV event identified on screening NWs using the indirect immunofluorescence antigen test (IFAT) (Light Diagnostics DFA Screen, Chemicon International Inc., Temecula, CA), an acute blood sample was collected as well as a convalescent sample 1 month later (See Figure 1 for schema of sample collection regime in relation to age and epidemics).

### Enzyme Linked Immunosorbent Assay (ELISA) Procedure

The enzyme-linked immunosorbent assay (ELISA) was based on that of Wilson *et al.* [23]. RSV laboratory strain A2 was used to infect HEp-2 cells. Following harvesting of lysate from both mock-infected and RSV-A2-infected cells, the resulting supernatant was sonicated (Sonics & Materials, Inc., Newton, CT) at 70% amplitude, 3×1 min cycles with 1s pulse and 1s pause. Lysates were then vortexed thoroughly prior to coating triplicate wells of 96-well Nunc-Immuno™ MaxiSorp™ plates (Fisher Scientific, Leicestershire, UK) with 25 µL of a 1/32 dilution in phosphate buffer saline (PBS) coating buffer of either RSV-A2-infected or mock-infected cell lysates. Plates were dried overnight at 37°C in a rotating incubator. These were then blocked with 200 µL/well of 5% dried milk (Marvel) in PBS and incubated for 1 hr at 37°C. Serum samples and controls were diluted 1/100 in dried milk in PBS. All plates included serial dilutions (1/50 to 1/1600) of a high titre local standard of pooled adult sera used to generate a standard curve. This was given an arbitrary unit (AU) value of 1000. Resultant test OD readings were adjusted for mock antigen results. All test sera were then calibrated using the standard curve and Ab reported as AU. The rest of the assay was carried out as previously described [23].

### Indirect immunofluorescence antigen test (IFAT)

This was carried out as previously described by Nokes *et al*. [21].

### Definitions

An ‘IFAT confirmed’ serological response was defined as a seroconversion or two-fold rising titre (0.3 log_10_AU) occurring between acute and convalescent sera collected approximately 1 month apart on identification of IFAT positive NWs. An ‘ELISA confirmed’ serological responses was defined as a 2-fold specific Ab increase occurring between two samples collected 3 months apart (without antigen confirmation by IFAT).

### Data Analysis

All data analysis was undertaken using Stata 9.0™. As Ab measurements were highly positively skewed, all analyses were carried out after the transformation of anti-RSV Ab serological levels from standardized AU to log_10_ AU. The determination of an appropriate cut-off delineating positive and negative sera was assessed using a frequency distribution of Ab titres for all sera screened. Seroconversion was defined as a 2-fold (0.3 log AU) or greater rise in titres between 2 samples collected consecutively. Passively acquired IgG is subject to an exponential decay rate [24], hence the rate of RSV-specific matAb decay, α, was estimated using the linear regression on log Ab concentrations, assuming
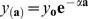
where *y*_o_ is the mean Ab level at birth and *y*_(a)_ the mean Ab titre at a given age, *a*.

Accounting for clustering due to multiple measurements per child was undertaken using a random effects model.

## Results

### Descriptive Analysis of study population

Six hundred and thirty five infants were recruited to the birth cohort and followed to approximately 3 years of age. A total of 521 cord bloods, 2,777 three monthly serum samples, 295 acute and 272 convalescent blood samples were collected (Figure 1). The male: female ratio was approximately 1∶1, with the majority of infants weighing between 2–3 kg (47.7%) or 3–4 kg (46.1%) at birth. The majority were either 1^st^ (28.7%), 2^nd^ (21.3%), 3^rd^ (15%) or 4^th^ (14.8%) born.

### Frequency distribution and prevalence of RSV-specific antibody titres

**Figure 2:**
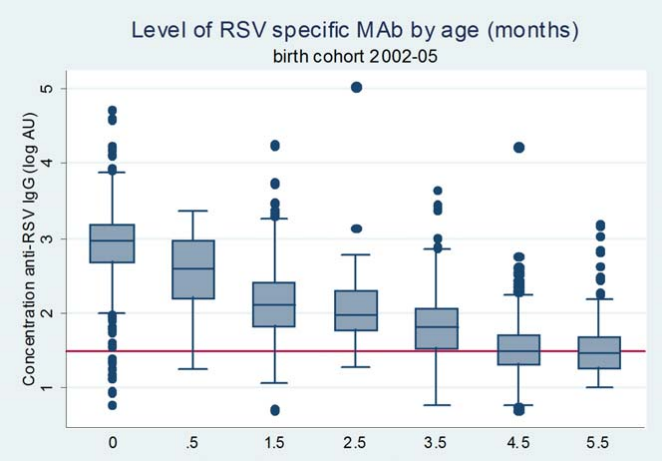
The level of RSV-specific matAb by age (months) in a birth cohort, Kilifi Kenya. Box plot of log_10_AU Ab levels for age categories defined as 0 months (cord), 0.5 (>0-<1), 1.5 (1-<2), 2.5 (2-<3), 3.5 (3-<4), 4.5 (4-<5) and 5.5 (5-<6) months. The red horizontal line defines the cut-off for seropositivity of 1.5 log_10_AU.

**Figure 3:**
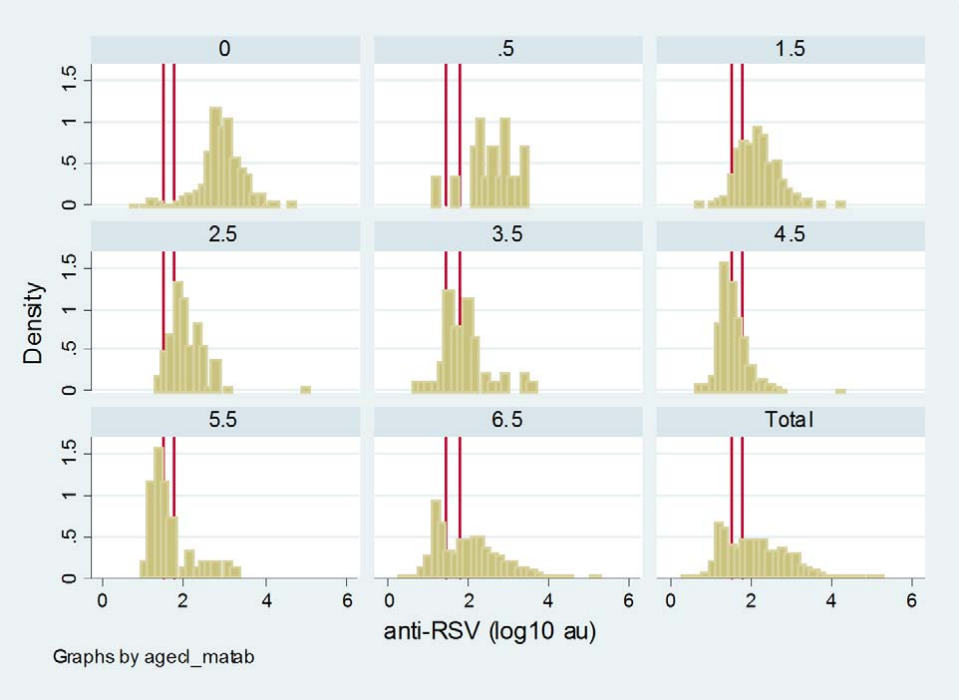
Frequency distribution of antibody titres (log_10_ AU) by age class (months). Cut-off between seropositive and seronegative is shown by red lines and lies between 1.5–1.8 (log_10_AU).

The RSV-specific Ab titres during the first 6 months are shown in Figure 2. A cut-off between seropositive and seronegative sera fell within the range of 1.5–1.8 log AU as noted from the bimodal frequency distribution of Ab titres for all sera and by age group (Figure 3). The mean Ab titre for seropositive infants at the cut-off of 1.5 log AU (also the lower limit of sensitivity of the ELISA - data not shown) was then constructed and indicated an average low titer point at about 4–5 months of age, with a low plateau maintained from about 4–10 months of age. A similar profile was noted when using the 1.8 log AU cut-off (this is the level at which approximately 50% of population was seronegative; profile not shown).

**Figure 4:**
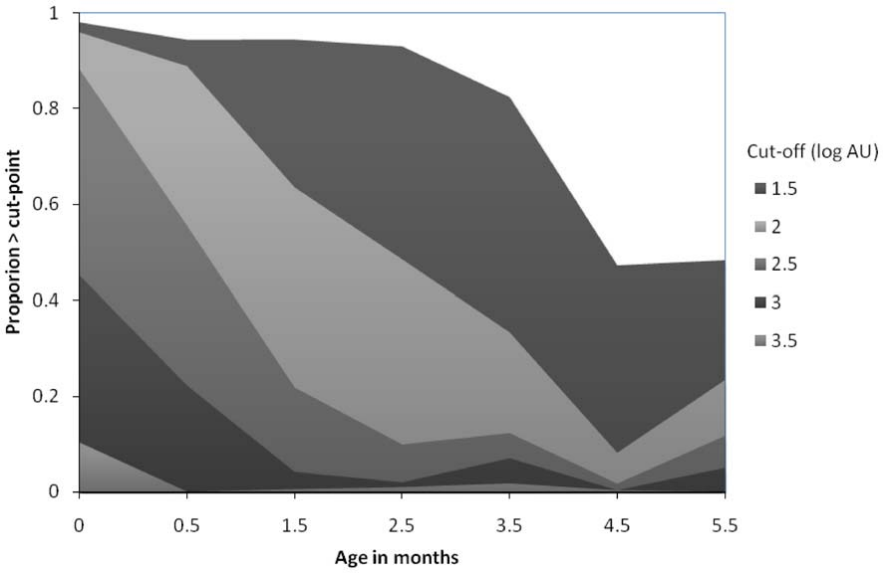
Age-seroprevalence profile at various cut-off levels of seropositivity. The assay cut-off value of seropositivity is 1.5.

We also compared the proportion of samples above the 1.5 log cut-off for positivity during 0–6 months of life (Figure 4). At the cut-off of 1.5 log AU, 97% of infant displayed matAb. Seroprevalence gradually declined with age to around 50% at ages 4-<5 and 5-<6. The effect of raising the cut-off for seropositivity between logAU 1.5 and 3.5 is shown in Figure 4. Seroprevalence is around 10% of children by age 4.5, 2.5, 1.5 and 0 months for progressively higher cut-off levels of 2, 2.5, 3, and 3.5, respectively.

### Analysis of the antibody response model

**Figure 5:**
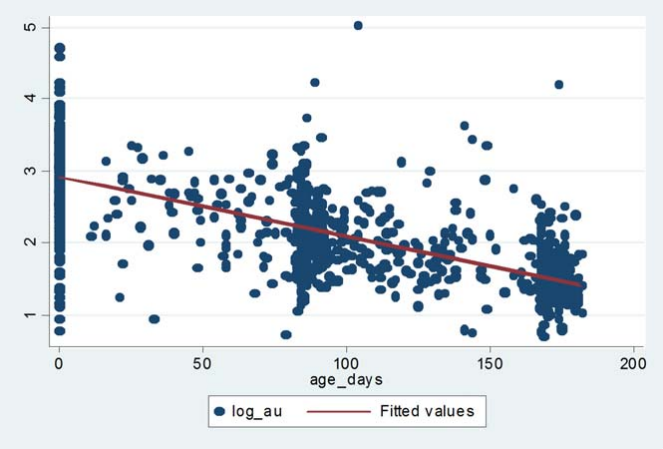
Scatter plot of Ab titres (log_10_ AU) during the first 6 months of life. The predicted line of fit by simple linear regression: *y* = mx+c (rate of decay (slope), m = −0.008; intercept, c = 2.928) is indicated. Only children without serologic evidence of infection during the first 6 months of life are depicted.

From the Ab titre distribution curve, it was noted that RSV-specific matAb declined log linearly over the first six months of life. A simple linear regression model was fitted through all the data points for children less than 6 months of age irrespective of whether they experienced any infection or not (Figure 5) and the rate of decay calculated. For Ab values<1.5 log AU these were corrected to 1.5 log AU, and the rate comprising these values compared to the rate when the latter were not corrected. No significant difference was noted. To calculate the ‘pristine’ rate of decay in the seropositive population, infants who experienced at least 1 infection, defined as either an ELISA-confirmed or IFAT-confirmed serological increase, were excluded. This gave 2 population groups; non-infected (851 samples) and infected (372 samples). A random effects mixed model to account for the multiple measurements per child was investigated and compared to the simpler linear model. No significant difference was found between the predictors used in these two models.

The ‘pristine’ half-life (T_1/2_) in days of RSV-specific matAb for the seropositive population was 79 days (95% CL: 76–81 days), whilst the mean ‘pristine’ duration or length of time that an infant remained above the cut-off (in days) was 112 (95% CL: 107–118). No significant differences were noted for either T_1/2_ or mean duration of RSV-matAb when the whole population (infected group included) was similarly analyzed.

### Examination of possible risk factors affecting cord blood levels and rate of decay

The effects of risk factors on cord blood levels of both infected and non-infected populations were evaluated initially using a two-sample *t-test* followed by a multiple linear regression model. With regards to matAb titres, after controlling for birth weight level, birth order levels and being born in or out of an epidemic, using a multiple linear regression model, a univariate analysis was initially performed, and subsequently all variables, irrespective of significance level at univariate analysis, combined. The matAb titres for the infected groups remained significantly lower (*P = 0.011*) when compared to the non-infected groups (3.02 log AU versus 2.81 log AU; *t = 2.32*, *df = 91.46*, *P = 0.011*; 1 tail; an ELISA-confirmed serological response at the at the 2-fold seroconversion cut-off level).

It was noted that only cord blood levels (*P<0.001*) affected the rate of decay of the two population subgroups (non-infected and infected children), the remaining risk factors having no effect. With reference to the rate of decay or differences in gradient of matAb decline between these two population subgroups however, no differences (−0.009 versus −0.007, *t = −1.47*, *df = 109.39*, *P = 0.145*; 2 tail) were identified.

## Discussion

RSV specific matAb and infection were investigated in a birth cohort comprising 635 children in Kilifi District. The distribution, duration and risk factors of RSV maternally-derived immunity over the 1^st^ six months of life were explored.

At the cut-off point for seropositivity of 1.5 log AU (ascertained from the bimodal distribution of the assay results), maternal transfer was deemed to be efficient as approximately 97% of infants displayed RSV-specific matAb at birth. By 6–7 months however, 50% infants were noted to be still seropositive. This is in contrast to earlier studies [6], [7], [25] with the exception of the study by Ebihara *et al*. [26], in which seroprevalence levels were seen to reach a nadir by 6 months of life, and seroprevalence varied from between 2–16% when carried out by the ELISA, and 21% by both the G- and F-specific (RSV surface glycoproteins) competition ELISA. In the study by Ebihara and colleagues [26], a similar level of prevalence of 48% was observed.

Increasing the cut-off levels for seropositivity from 1.5 to 1.8 log AU and above, resulted in a more rapid decline in seropositivity and a minimum Ab level being attained by 6 months, as previously described [6], [7], [25]. There is little data to indicate what level of systemic anti-RSV Ab provides adequate transudate across the respiratory mucosa to confer protection from infection. The relationship between cut-off level and rate of decay in seroprevalence (Figure 4) provides possible scenarios for the rate of decay of actual protective titres of RSV antibody which requires clarification.

It should be borne in mind that the measurement of ELISA binding titres using a crude extract is an indirect measure of functional Ab. It would have been more suitable to measure neutralizing Ab titres to get a direct measurement of functional Ab following infection and in the presence of matAb. Furthermore, the use of A2 rather than the currently circulating strain in the assay procedure could have led to an under-estimation of seroconversion due to poor cross-reactivity [4].

Over the first 6 months, the ‘pristine’ T_1/2_ of RSV-specific matAb in the population without both serologic evidence and/or IFAT-positive indication of infection was estimated as 79 days (95% CL: 76–81) and matAb had an average duration of 112 days (95% CL: 107–118). This rate of decay is shorter than that observed by Cox *et al*. [7], Ward *et al.* [15] and Hacimustafaoglu *et al*. [25]. These authors calculated rates that varied from between 91–100 days, although it is not clear as to whether the authors included or excluded seronegative individuals with infections in their calculations. This rate for RSV- matAb decay for the Kilifi population however, was longer than that for measles, mumps and rubella [24], [27], and human parainfluenza type 3 [28], which vary from 35–40 and 51 days respectively. Again, however, it is important to recognise the likely difference between the protective potential of matAb to these systemic infections as opposed to protection against RSV at the respiratory mucosa.

Our study suggests that the rate of matAb decay does not differ between the wider population that also included those who had infections compared to the non-infected population. This implies that RSV infections below 6 months of age have no significant effect on the rate of RSV-specific matAb decay. In other words, the presence of matAb has a masking effect on infections under 6 months of life, and thus seroconversions in this age group will not be easily identified. Risk factors affecting both RSV cord titres and RSV matAb decline were analyzed using both the 2-sample t-test and multiple linear regression analysis. With respect to cord blood titres, children who went on to experience an infection within 6 months of age were observed to have consistently significantly lower titres in comparison to the children who did not experience infection within this period. This is in agreement with the study by Stensballe and others [29], who found a clear correlation with decrease in mean cord blood RSV Ab titres and steep increase in the number of RSV hospitalizations in infants younger than 6 months of age. This therefore implies that matAb are protective as earlier described [2], [12], [14], which has implications for the development of a maternal vaccine [2], [25].

The relatively short half-life of RSV matAb of about 2.5 months suggests that a childhood vaccine could be administered fairly soon after this, assuming minimal matAb interference. However, at least 50% of the population remain seropositive at 4–5 months of age, and it is plausible that existing Ab titres could interfere with vaccine response and hence childhood vaccines may not be useful in this setting, or a schedule with later boosting should be considered. Delay in the age at vaccine delivery would however fail to prevent many infections that were observed to occur in this age group. As high levels of matAb are known to be protective, maternal vaccination might be another option in this population. Apart from reducing the potential risk of infection to the mother and hence the child, a maternal vaccine would potentially boost matAb levels when transferred to the infant via transplacental transfer or through breast-feeding, protecting the infant during the vulnerable period of life when their immune system is still under-developed. A clear association between maternally derived RSV neutralizing antibody titre and RSV seasonality in infants younger than 6 months of age has recently been described [29]. It thus remains important that the critical vaccination fraction (those fractions of each subpopulation that should be vaccinated to achieve protection against RSV) as well as the recommended age for vaccination be better established, especially within the developing country setting.
